# 

**Published:** 1865

**Authors:** 


### INDEX OF HALL'S JOURNAL OF HEALTH.

### VOL. XII, 1865.

PAGE

Aphorisms Physiological, 65

Apples, 58, 115

Antidote to Poisons, 134

Acre, One, 139

Apoplexy, 201

Burying Alive, 4

Baths and Bathing, 30

Beards, 74

Bites and Burns, 23, 80

Backbone, 91

Beauty a Medicine, 106

Best Day, 119

Baldness, 124

Burning to Death, 129

Bilious Diarrhea, 153

Balm of Gilead, 198

Bread, 193, 210

Cold Cured, 3

" Neglected, 29

" Avoided, 31

" Nothing but a, 36

" In the Head, 52

" How Taken, 59

" Catching, 144

Consumption, 16, 19, 116

Coffee-Drinking, 46, 72, 128

Catarrh, 52

Checking Perspiration, 59

Centenarian, 76

Child-Bearing, 92

Children's Eating, 118

" Feet, 180

Children Corrected, 187

" Dirty, 225

Coal-Fires, 120

Cute Things, 126

Coffee Poisons, 128

Clothing, Flannel, 119

" Woolen, 130

" Changing, 141

Cholera, 151

Clergymen, 158

Cancer, 177

Corn-Bread, 84, 185

Convenient Knowledge, 188

Charms, 190

Cooking Meats, 205

Cheap Bread, 210

Church Ventilation, 229

Cough, 230

Dyspepsia, 8, 33

Drinking, 35

PAGE

Diet for the Sick, 54
Dieting, 86, 147
Deafness, 70, 121
Debt, a Death! 108
Diarrhea, 97, 150, 152, 153
Death, 4, 88, 110, 129, 143, 146, 159
Dying Easily, 143
Drowning, 146
Diphtheria, 149
Dysentery, 152
Disinfectants, 154
Death Rate, 159
Deranged, 177, 186, 222, 233
Digestibility of Food, 218
Dirty Children, 225
Drugs and Druggery, 231
Eyes, Care of, 5, 15
" Weak, 15
" Failing, 61
Erect Position, 11
Eating, 26
" Wisely, 32
Eat, How to, 34
" What and When to, 40
Eating Habits, 142
" Great, 171
" Curiosities of, 191
" Economical, 196
Emanations, 195
Elements of Food, 223
Fruits, Uses of, 2
Flannel Wearing, 19
Follies, Fifteen, 53
Fire-Places, 114
Fifth Avenue Sights, 163
Food and Health, 206
Feet, 21, 180, 216
Fetid Feet, 221
Food, Nutritiousness of, 219
" its Elements, 223
Greed of Gold, 102
Genius, Vices of, 161
Great Eaters, 177
Gruels and Soups, 181
Hair Wash, 18
Health a Duty, 20
" Observances, 38
" Essentials, 42
" Theories, 71
Hydrophobia, 49
Headache, 41, 62
Housekeeping, 75, 94, 192

| | PAGE |
|---|---|
| Housewifery, | 192 |
| Household Knowledge, | 203 |
| Habit, | 99 |
| Home, Leaving, | 162 |
| Happiest, Who are, | 204 |
| Hunger, | 217 |
| Horse Rations, | 222 |
| Inconsiderations, | 1 |
| Ice, Uses of, | 9 |
| Inverted Toe-Nail, | 102 |
| Insanity, | 173, 186 |
| In the Mind, | 189 |
| Kindness Rewarded, | 184 |
| Law of Love, | 89 |
| Longevity, | 95 |
| Life Wasted, | 133 |
| Loose Bowels, | 97, 150 |
| Leaving Home, | 162 |
| Logic Run Mad, | 172 |
| Medicine Taking, | 60 |
| Memories, | 75 |
| Music Healthful, | 7, 81 |
| Milk, its Uses, | 82 |
| Miasm, | 98 |
| Marriage, | 105 |
| Morning Prayer, | 113 |
| Month, Malign, | 170 |
| Mental Ailments, | 189 |
| Mind Lost, | 222 |
| Medical Science, | 227 |
| Neuralgia, | 44 |
| Nursing Hints, | 57 |
| Nervous Debilities, | 63 |
| Old Age Beautiful, | 7, 15 |
| One Acre, | 139 |
| One by One, | 160 |
| Obscure Diseases, | 165 |
| Precautions, | 37 |
| Presence of Mind, | 39 |
| Premonitions, | 43 |
| Private Things, | 45 |
| Poisons and Antidotes, | 134, 55 |
| Pain, | 67 |
| Peaceless, | 83 |
| Preserves, | 103 |
| Parental Trainings, | 108 |
| Philosophy, | 137 |
| Physiological Items, | 155 |
| Posture in Worship, | 176 |
| Physician, Faithless, | 197 |
| Popular Fallacies, | 228 |
| Punctuality, | 234 |
| Providence, | 235 |
| Rheumatism, | 50 |
| Read and Heed, | 69 |
| Resignation, | 145 |
| Restless Nights, | 208 |
| Recreation, Summer, | 209 |
| Restlessness, | 233 |
| Sitting Erectly, | 13 |
| Shoes Fitting, | 14 |
| Sabbath, | 17, 85, 104, 119, 156 |
| Sour Stomach, | 24 |
| Sick Headache, | 41 |
| Sunshine, | 51 |
| Skating, | 63 |
| Suppers, Hearty, | 73 |
| Soldiers Remembered, | 90 |
| " Cared for, | 100 |
| " Health, | 111 |
| " Items, | 117 |
| " All, | 112 |
| Serenity, | 96 |
| Sores, | 101 |
| Sunday Dinners, | 104 |
| Small-Pox, | 107 |
| Sleep and Death, | 110 |
| Spot the One, | 127 |
| Specifics, | 138 |
| Spring-Time, | 140 |
| Summer Drinks, | 148 |
| Sickness not Causeless, | 164 |
| Sayre, the Banker, | 166 |
| September Malign, | 170 |
| Summer Mortality, | 175 |
| Stammering, | 179 |
| Soups and Gruels, | 181 |
| Sick School-Girl, | 183 |
| Stomach's Appeal, | 202 |
| Sleep, | 25, 110, 208 |
| Summerings, | 79, 75, 148, 209 |
| Study, Where to, | 227 |
| Salt Rheum, | 226 |
| Sordid, | 232 |
| Traveling Hints, | 6 |
| Three Ps, | 47 |
| Teeth, | 68 |
| Toe-Nail, Inverted, | 102 |
| Thankful Ever, | 236 |
| Urination, | 66 |
| Valuable Knowledge, | 27 |
| Vaccination, | 78 |
| Ventilation, | 125 |
| Vermin Riddance, | 136 |
| Winter Rules, | 10 |
| Walking, | 12 |
| Warning Youth, | 48 |
| Woman's Beauty, | 87 |
| Whitlow, | 109 |
| Whitewashes, | 131 |
| Worth Remembering, | 167 |
| Worship, Public, | 168, 176, 198 |
| " Posture in, | 176 |
| " Without Price, | 198 |
| Weather Signs, | 178 |
| Weather and Wealth, | 200 |
| Warmth and Strength, | 220 |
| Worth Knowing, | 224 |

